# Monoclonal Antibody to CD14, TLR4, or CD11b: Impact of Epitope and Isotype Specificity on ROS Generation by Human Granulocytes and Monocytes

**DOI:** 10.1155/2020/5708692

**Published:** 2020-11-20

**Authors:** Dmitry S. Kabanov, Sergey V. Grachev, Isabella R. Prokhorenko

**Affiliations:** ^1^Department of Molecular Biomedicine, Institute of Basic Biological Problems, Federal Research Center “Pushchino Scientific Center for Biological Research of the Russian Academy of Sciences”, Pushchino 142290, Russia; ^2^Department of Human Pathology of the Institute of Clinical Medicine, Federal State Autonomous Educational Institution of Higher Education I. M. Sechenov's First Moscow State Medical University of Russian Healthcare Ministry (Sechenov University), Moscow 119991, Russia

## Abstract

Lipopolysaccharides (LPSs or endotoxins) from Gram-negative bacteria represent pathogen-associated molecular patterns (PAMPs) that are recognized by CD14 and Toll-like receptor 4 (TLR4). Lipopolysaccharides prime polymorphonuclear leukocytes (PMNs) for substantial production of reactive oxygen species (ROS) during its response to secondary stimuli such as chemoattractants or pathogens. The excessive ROS production can damage surrounding host tissues, thereby amplifying the inflammatory reaction caused by pathogens. Today, specific antibodies against CD14, TLR4, and CD11b are being used as the essential tools to elucidate the role of these receptors in acute inflammation and some of these antibodies have advised as therapeutic agents for clinical use. Because each antibody has two antigen-binding arms [F(ab′)_2_] and one Fc arm, its effect on cellular response is much more complicated rather than simple blockage of target receptor. In fact, IgG antibody, once bound to target receptor, engages Fc receptors *γ* (Fc*γ*Rs) and thereby is able to activate the adaptive immune system. The consequences of antibody-dependent binary heterotypic association of CD14, TLR4, or CD11b with Fc*γ*Rs as well as homotypic one on ROS production are not well elucidated. Moreover, the consequences of antigenic recognition of CD14, TLR4, or CD11b by specific F(ab′)_2_ fragments are not always investigated. In this review, we will discuss known mechanisms underlying the therapeutic efficiency of CD14, TLR4, and CD11b/CD18 antibodies with a focus on LPS-dependent ROS or cytokine production by PMNs or monocytes. The impacts of F(ab′)_2_ as well as antibody IgG subclasses (isotypes) in therapeutic efficiency or agonistic potency of known antibodies against abovementioned receptors are presented. We also pay attention to how the efficiency of different IgG antibody subclasses is modulated during LPS-induced inflammation and by production of priming agents such as interferon *γ* (IFN-*γ*). Our review reinforces the molecular targets and therapeutic approaches to amelioration of harmful consequences of excessive activation of human pattern recognition receptors.

## 1. Introduction

Inflammatory and immune diseases affect millions of people worldwide, providing an impetus to develop new anti-inflammatory and immunomodulatory therapies. Over the past two decades, great progress has been made in elucidating the molecular basis of the inflammation process during infectious, autoimmune, and malignant diseases [1–4]. It favors the development of new therapeutic drugs directly targeting cell surface or intracellular molecules involved in the initiation and progression of inflammation. In the case of endotoxemia, the major attention has been on application of LPS analogs with “under-acylated” lipid A structures, synthetic nontoxic lipid A derivatives, monoclonal antibodies to lipid A or truncated Re-LPS structure, blocking antibodies against both cell surface receptors and cytokines, and to other intracellular small molecule antagonists for therapeutic purposes. Today, a range of specific antibodies against CD14, TLR4, and CD11b are being used as the essential tool to elucidate their role in acute inflammation [1, 5–16]. We have shown earlier that some certain antibodies such as anti-CD11b^ICRF-44^Fc^mIgG1^ or anti-TLR4^HTA125^Fc^mIgG2a^ are unable to ameliorate significantly the *N*-formyl-methionyl-leucyl-phenylalanine- (fMLP-) triggered ROS production (luminol) from LPS-primed PMNs [12, 13], while anti-CD14^UCHM-1^Fc^mIgG2a^ suppresses LPS priming successfully [14]. Molecular mechanisms underlying our observations, however, have not been described. So, in this review, we discuss molecular mechanisms underlying LPS-induced functional responses of human PMNs and monocytes/macrophages such as ROS generation and production of cytokines after cell exposure to mouse IgG (mIgG) antibody to CD14, TLR4, or CD11b.

ROS is a collective term that often includes not only superoxide anion radical (O_2_^·−^) but other oxygen radicals such as hydroxyl (OH^−^), peroxyl (RO_2_), alkoxyl (RO^·^), hydroperoxyl (HO_2_^·^), and also nonradicals as hydrogen peroxide (H_2_O_2_), hypochlorous acid (HOCl), singlet oxygen (∆gO_2_), and peroxynitrite (ONOO^−^) [17]. Among them, H_2_O_2_ is relatively stable diffusible oxidant acting as a signaling molecule and second messenger in the inflammatory settings. Today, it is known that signaling or damaging actions of ROS are determined by its amount, type, and cellular location. For example, H_2_O_2_ has been shown to be involved in activation of nuclear factor NF-*κ*B and probably in MAPK signaling cascades [18–22].

Circulating leukocytes are programmed for distinct functions in human physiology. The three main antimicrobial functions are accepted for PMNs: phagocytosis, degranulation, and the release of nuclear material in the form of PMN extracellular traps. Nowadays, it is recognized that PMNs can produce cytokines, modulate activities of neighboring cells, contribute to the resolution of inflammation, regulate macrophages for long-term immune responses, and even have a role in innate memory [23, 24]. The main function of monocytes is the “processing” and degradation of antigens. Once produced from the bone marrow into the blood, circulating monocytes should be rapidly activated by inflammatory signals and migrate to areas of inflammation where they can differentiate into the proinflammatory (M1) or anti-inflammatory (M2) phenotypes known as tissues macrophages. In the M1 state, the activated monocyte-macrophages undergo a metabolic switch from the oxidative phosphorylation to glycolysis. Inhibition of oxidative phosphorylation increases ROS production which exerts bactericidal activities. During the resolution of inflammation, abundance of anti-inflammatory (M2) monocyte-macrophages with more oxidative phosphorylation phenotype is increased [25]. Classically activated M1 monocyte-macrophages have elevated microbicidal function associated with the ability to secrete high amount of proinflammatory cytokines (TNF-*α*, IL-1*β*, and IL-12) and ROS, while alternatively activated M2 monocyte-macrophages produce high levels of anti-inflammatory mediators (IL-10 and TGF-*β*) [26].

A change in redox homeostasis may facilitate differentiation of monocytes into macrophages [26]. In fact, in human myeloid leukemia PLB-985 cells, during VD3-triggered monocyte-to-macrophage differentiation, the expression and translocation of nicotinamide adenine dinucleotide phosphate (NADPH) oxidase components to the plasma membrane coincides with upregulation of surface markers such as CD11b and CD36 [26, 27]. Mitochondrial ROS (mitoROS) contribute to LPS-induced cytokine release by monocyte-macrophages [18, 28]. For example, mitoROS regulate IL-1*β* transcription (inflammasome priming), but may also regulate the maturation and secretion of IL-1*β* (inflammasome activation) [18, 29].

ROS are by-products of numerous enzymatic reactions in various cell compartments, including the cytoplasm, cell membrane, endoplasmic reticulum, mitochondria, and peroxisome [18]. It has been suggested that peripheral blood monocytes depend on oxidative phosphorylation (ATP synthesis) for their energy supply, whereas PMNs do not. PMNs lose their mitochondrial dependency during maturation from bone marrow mitochondrion-rich precursors into peripheral blood PMNs with relative few mitochondria [30]. As the result, it has been proposed that mitochondria in PMNs (unlike monocyte-macrophages) do not play a role in energy metabolism, but maintain mitochondrial membrane potential for apoptotic signaling [25]. PMNs during phagocytosis use large quantities of molecular O_2_ not for mitochondrial respiration, but, rather, to generate O_2_^·−^ and other oxidants via a respiratory burst catalyzed by NADPH oxidase [31].

NADPH oxidase is the enzyme responsible for O_2_^·−^ production [32]. This multicomponent enzyme system is composed of two transmembrane proteins (p22^phox^ and gp91^phox^/NOX2 forming cytochrome b_558_), three cytosolic proteins (p40^phox^, p47^phox^, and p67^phox^), and GTPase (Rac1 or Rac2). These components of NADPH oxidase are assembled at membrane sites upon transition of PMNs to a state of enhanced responsiveness known as priming. Three major events accompany activation of NADPH oxidase: (1) protein phosphorylation, (2) activation of GTPases, and (3) translocation of cytosolic components of NADPH oxidase to plasma membrane or to membrane of intracellular granules. Actually, NADPH oxidase in PMNs exists in different states: resting, primed, activated, or inactivated [33]. It has been demonstrated that O_2_^·−^/ROS derived by NADPH oxidase are critically involved in LPS intracellular signaling leading to PMN priming as well as to maintenance of the resting or nonprimed state [34–37]. The primed PMNs have been identified in humans with infections, rheumatoid arthritis, chronic kidney disease, traumatic injury, and acute respiratory distress syndrome [38].

As known, PMNs express a range of receptors including *β*_2_ integrins (CD11/CD18) and Fc receptors *γ* (Fc*γ*Rs) which are capable of initiating complex intracellular signaling events robustly activating NADPH oxidase. In addition, some members of G protein-coupled receptors (GPCRs), especially fMLP receptor FPR1, can directly activate NADPH oxidase, although to a lesser extent than what has been observed with activated integrins or Fc*γ*Rs [32]. It is necessary to note that LPS itself does not elicit in PMN significant O_2_^·−^/ROS production but transforms them into a primed state in which NADPH oxidase is not fully assembled but becomes more susceptible to activation by secondary stimuli [32–34, 39, 40].

## 2. TLR4 and Their Intracellular Signaling Molecules

LPS in the bloodstream is recognized by LPS-binding protein (LBP) that transfers them to CD14 followed by their presentation to MD-2·TLR4 on the surface of monocytes and PMNs [1, 5, 6, 11, 41–43]. Structural LPS-induced rearrangements in MD-2·TLR4 trigger TLR4 partitioning into lipid rafts where it undergoes homotypic dimerization facilitating signal transduction events. TLR4 operates with the assistance of other cell surface receptors which are assembled in the LPS-induced “receptor cluster” [6]. Besides CD14 and TLR4, other receptors including the *β*_2_ integrin CD11b/CD18 and Fc*γ*Rs (CD16A, CD32, and CD64) have been also detected as constituents of monocyte LPS-induced “receptor cluster” [41, 42, 44].

MyD88-dependent and MyD88-independent TRIF-dependent signaling pathways have been described in monocytes following TLR4 activation [45, 46]. These signaling pathways are dependent on Toll/interleukin-1 adaptor proteins including MyD88, TIRAP/MAL, TRIF/TICAM-1, and TRAM/TICAM-2 [47–49]. It has been shown that the LPS-caused initiation of MyD88-dependent pathway results in rapid NF-*κ*B activation and release of proinflammatory cytokines (TNF-*α*, IL-1*β*, and IL-6) and chemokines (MCP-1, MIP-3*α*, and IL-8). Moreover, in monocytes, the LPS-caused initiation of the MyD88-independent pathway results in rapid activation of interferon regulatory factor 3 (IRF3) leading to release of beta interferon (IFN-*β*) and to the second delayed NF-*κ*B activation [50, 51]. Unlike monocytes, the MyD88-independent signaling pathway cannot be mobilized in PMNs in the response to LPS [52].

An amplified O_2_^·−^/ROS production from LPS-primed and fMLP-stimulated PMNs is the result at least of two converging intracellular signaling pathways. The first LPS-induced signaling pathway engages in PMNs such adaptor proteins as MyD88, TIRAP/MAL, IRAK, TRAF6, and TAK1. Among them, TAK1 is linked to MAPK signaling cascades [52]. After 20 minutes of PMN exposure to LPS, the MKK3-dependent phosphorylation of p38 MAPK is observed [53]. The p38 MAPK-dependent translocation of cytochrome b_558_ and p47^phox^ but not p67^phox^ or Rac2 to the plasma membrane is also known. fMLP in LPS-primed PMNs causes a rapid and strong translocation of the other cytosolic components of NADPH oxidase to the already mobilized cytochrome b_558_ followed by O_2_^·−^/ROS production [54].

## 3. Heterotrimeric G_i*α*2_ Proteins and Their Intracellular Signaling Events

The second fMLP-initiated pathway is realized by FPR1 coupled with heterotrimeric G_i*α*2_ proteins. The activated G_*βγ*_ subunit of G_i*α*2_ initiates concomitant activation of phospholipase C (PLC) and PI3K signaling pathways. The activity of p38 MAPK and ERK1/2 kinases is also upregulated during activation of G proteins [55–57]. Activated PLC hydrolyses phosphatidylinositol 4,5-bis-phosphate [PtdIns(4,5)P_2_ or PI(4,5)P_2_] in the plasma membrane leading to production of inositol 1,4,5-triphosphate [Ins(1,4,5)P_3_] that is followed by Ca^2+^ release from intracellular stores and generation of diacylglycerol (DAG), which in turn activates protein kinase C (PKC). The increase in intracellular free Ca^2+^ leads to Ca^2+^ influx into the cell. A rise in Ca^2+^ is an essential step in PMN activation and O_2_^·−^/ROS generation. Activated PKC induces phosphorylation of several substrates including p47^phox^ of NADPH oxidase. At the same time, activated PI3K produces phosphatidylinositol 3,4,5-triphosphate [PtdIns(3,4,5)P_3_ or PI(3,4,5)P_3_] from PtdIns(4,5)P_2_. The ability of wortmannin to inhibit PI3K and to abolish the fMLP-triggered respiratory burst without any effect on agonist-induced [Ca^2+^]_*i*_ flux or PKC-mediated NADPH oxidase activation has provided strong evidence to support a second-messenger role for PtdIns(3,4,5)P_3_ in O_2_^·−^ generation [58].

In monocytes, LPS-induced release of proinflammatory cytokines is mediated by PI3K in both a ROS- and G protein-dependent manner, propagated through NADPH oxidase complex 4 (NOX4). Upregulation of PKB/Akt is completely inhibited by pretreatment of human PBMC with either pertussis toxin (inhibitor of G_*α*i_PCRs) or apocynin (inhibitor of NADPH oxidase 4) [21].

## 4. Human Fc*γ* Receptors and Their Ligands

Monoclonal antibodies to cell surface receptors such as CD14, TLR4, or CD11b/CD18 have various modes of actions. The simplest mode of their action is mere binding of the antibody to its antigen, thereby interfering or not with receptor activation. On the other hand, the antibody is able to block receptor interaction with their ligand, interfering with a multimerization process or triggering internalization of the receptor. In addition, once bound to antigen, IgG antibodies can engage the adaptive immune system via the interaction of their constant Fc region with Fc*γ*Rs [59]. The human Fc*γ*R family contains six known members in three subgroups, including CD64, CD32 (CD32A, B, C), and CD16 (CD16A, B) [60]. CD32A is mainly expressed on monocytes (5 × 10^4^/cell), macrophages, and PMNs (1–4 × 10^4^/cell), whereas CD32B is on B cells principally [11, 61–63]. The cytoplasmic domain of CD32A contains the immunoreceptor tyrosine-based activation motif (ITAM), while CD32B the immunoreceptor tyrosine-based inhibitory motif (ITIM) [11]. Human CD64 is abundantly expressed on monocytes (15–40 × 10^3^/cell) while at lower levels on PMNs (1–2 × 10^3^/cell) and macrophages (1 × 10^5^/cell) [61, 62]. Human CD64 could be engaged by human IgG1 or mouse IgG2a (mIgG2a) but not mIgG1 or mIgG2b. Human CD32 appears to be engaged by mIgG1 or mIgG2b preferentially [64–70]. It is becoming increasingly evident that many receptors on myeloid cells do not act in isolation, but rather cooperate with other receptors to coordinate responses to stimuli. For example, during immune complex (IC) recognition by the cell, CD11b/CD18 cooperates with CD16B (1–3 × 10^5^/PMN) to cause Ca^2+^ flux and ROS generation. CD16 and CD11b/CD18 jointly prime CD32 for ROS generation [71, 72]. In human PMNs, both CD32 and CD16B are able to upregulate PI3K activity. Moreover, simultaneously engaged CD32 and CD16B via “inside-out” signaling can recruit and activate CD11b/CD18 on PMNs. Thus, three different types of interaction between Fc*γ*Rs and integrins could be realized: (1) a physical interaction on the cell surface, (2) integrin response that occurs because of Fc*γ*Rs engagement and “inside-out” signalization, and (3) cellular responses to Fc*γ*Rs that occur only after integrin occupation or when both receptors are stimulated simultaneously, i.e., “outside-in” signalization [73].

CD11b is able to regulate PtdIns(4,5)P_2_ generation at the cell membrane through an ADP-ribosylation factor (ARF6)-PIP5K pathway. The increase in PtdIns(4,5)P_2_ levels causes association of adaptor protein TIRAP/MAL with the plasma membrane, where it is needed to recruit MyD88 to TLR4 [74]. The functional coupling of aggregated CD64 to the PLD- and PKC-dependent activation of NADPH oxidase in IFN-*γ*-primed and IC-stimulated human monocytic U937 cells has been shown earlier. On the other hand, CD32A is coupled to PLC but is independent of PLD activation [75].

## 5. CD14-Associated Intracellular Signaling Events

CD14 is the most excessively studied TLR4 gatekeeper. Because CD14 is a glycosylphosphatidylinositol- (GPI-) anchored membrane protein without a transmembrane sequence, it is believed that CD14 has no intrinsic signaling ability during LPS recognition by innate immune cells. However, the LBP·LPS complex initially binds to CD14 and only then LPS is presented to the MD-2·TLR4 complex. CD14 controls the generation of PtdIns(4,5)P_2_ that is required for maximal LPS-induced TLR4-dependent proinflammatory signaling [76]. Moreover, CD14 is essential for LPS-dependent activation of phospholipases and MAPKs [77]. All together, these facts indicate CD14 as a promising therapeutic target. The impact of CD14 in TLR4-initiated signaling events has been studied in several works [64, 78–82] including our own [14].

### 5.1. CD14 in Ca^2+^ Signaling

Targeting CD14 by whole anti-CD14^Mo2^Fc^mIgM^ antibody is not able to stimulate Ca^2+^ mobilization in human PMNs (CD14 2–4 × 10^3^/cell) [83, 84]. However, in human monocytes, targeting CD14 (10–135 × 10^3^/cell) by whole anti-CD14^UCHM-1^Fc^mIgG2a^ antibody (divalent-Fc format) causes a rapid Ca^2+^ mobilization [64, 84]. This rise in intracellular free Ca^2+^ is less marked than that seen in the response to anti-CD32^CIKM5^Fc^mIgG1^ antibody [64, 78]. Similar to anti-CD14^Mo2^Fc^mIgM^ antibody, the antigenic recognition of CD14 by anti-CD14^UCHM-1^ F(ab′)_2_ fragments (divalent Fab_2_ format) does not elicit in human monocytes a raise in intracellular free Ca^2+^ [78]. Thus, in monocytes, Ca^2+^ signaling could be induced by antibody-dependent association of CD14 with the high affinity receptor CD64. In addition, antibody-dependent homotypic CD32 association (CD32 ← anti-CD32^CIKM5^Fc^mIgG1^ → CD32) is also able to induce Ca^2+^ mobilization. However, association of two CD32 is less effective for Ca^2+^ mobilization when compared to heterotypic CD14 association with CD64. When CD64 is saturated, the lower affinity CD32A may also be engaged by mIgG2a antibody (CD14 ← anti-CD14^UCHM-1^Fc^mIgG2a^ → CD64/CD32). It is necessary to note that anti-CD14^UCHM-1^Fc^mIgG2a^-induced Ca^2+^ mobilization is weaker than that caused by fMLP [64]. Thus, G protein-coupled FPR1 appeared to be a more potent inductor of Ca^2+^ signaling than the engagement of CD32 or CD64 (note not clustering).

Unexpectedly, Ca^2+^ mobilization in monocytes exposed to anti-CD14^UCHM-1^Fc^mIgG2a^ antibody has been not associated with O_2_^·−^ generation (SOD-inhibitable ferricytochrome C reduction) [64]. Although in our settings the anti-CD14^UCHM-1^Fc^mIgG2a^ antibody caused certain priming effect on fMLP-triggered O_2_^·−^/ROS production by human PMNs, we did not observe any statistically significant differences [14]. The data from other works have suggested that anti-CD14^UCHM-1^Fc^mIgG2a^ antibody is able to elicit in monocytes or PMN sufficient signal for phosphoinositide breakdown and Ca^2+^ mobilization but it is not enough to initiate the assembly of NADPH oxidase and O_2_^·−^/ROS generation [14, 78]. Sufficient mobilization of Ca^2+^ in all monocytes but only in subset of PMN (40%) has been detected only after CD14 crosslinking by anti-CD14^Mo2^Fc^mIgM^ or anti-CD14^MEM-18/63D3^Fc^mIgG1^ antibodies followed by secondary F(ab′)_2_ fragments. The broad homotypic aggregation (crosslinking) of CD14 in the plane of plasma membrane has been suggested to be responsible for the robust increase in H_2_O_2_/ROS production in monocytes while less pronounced in PMNs [80, 81]. The higher sensitivity of monocytes to antibody-dependent initiation of Ca^2+^ signaling in comparison to PMNs can be explained by the differences in CD14 levels on their cell surfaces [84].

The Ca^2+^ mobilization induced by CD14 crosslinking is suppressed when PLC or protein tyrosine kinases (PTK) have been inhibited [80]. Thus, only broad CD14 aggregation is able to stimulate substantial rise in intracellular free Ca^2+^ and O_2_^·−^/ROS production. CD14 in monocytes is physically associated with nonreceptor PTK ^Src^Lyn^p53/56^. The crosslinking of CD14 leads to ^Src^Lyn^p53/56^ activation followed by concomitant upregulation of ^Src^Frg^p58^ and ^Src^Hck^p59/61^ kinases [85, 86]. Earlier studies have shown that the signaling events triggered by CD14 crosslinking were abolished when the GPI anchor had been replaced by transmembrane sequence, suggesting that the localization to lipid rafts endowed CD14 with signaling ability [79, 80]. As GPI-anchored receptors have high lateral mobility in the plane of cell membrane, they may be more easily aggregated upon interaction with a specific ligand [81]. Thus, CD14 would function to concentrate LPS at the cell surface for their recognition by other LPS-binding proteins and to facilitation of PtdIns(4,5)P_2_ generation [87].

## 6. Epitope Specificity and Effectiveness of Anti-CD14 Antibodies against LPS-Induced Effects

LPS-binding sites on CD14 have been intensively studied, and four regions within the NH_2_-terminal 65 amino acid residues are identified. All of these regions (R) are clustered around the hydrophobic pocket of CD14. R1 (D_9_DED_12_) is located close to the wall, whereas R3 (A_35_VEVE_39_) is at the bottom of the pocket, while R2 (P_22_QPD_25_) and R4 (D_57_ADPRQY_63_) are located at the rim of the pocket. Another three regions of CD14, namely, T1 (E_7_LDDEDF_13_), T2 (L_91_RVLAYSRLKE_101_), and T3 (P_185_GL), have been proposed to be involved in LPS transfer to MD-2·TLR4 and therefore are responsible for LPS signaling. R1 within CD14 overlaps with T1 region. Therefore, the T1/R1 sequence appears to play a role in both LPS binding and transfer (LPS signaling) to MD-2·TLR4 [88, 89]. The effectiveness of various anti-CD14 antibodies against LPS-induced effects is listed in Table 1.

### 6.1. Targeting R1 and T1 by 3C10 Antibodies Interferes with LBP·LPS Binding to CD14

Anti-CD14^3C10^Fc^mIgG2b^ antibody binds to mostly anionic E_7_LDDEDFR_14_ sequence in CD14 that is able to interact with cationic proteins such as serum LBP [106, 111, 112]. Anti-CD14^3C10^ antibody almost completely prevents PMN priming by LBP·LPS for fMLP-triggered O_2_^·−^/ROS production (luminol) [90]. So, the first antibody-dependent mechanism downregulating LPS deleterious effects is based on the ability of anti-CD14 antibodies to prevent the binding of LBP·LPS to CD14 and to abolish subsequent LPS transfer to MD-2·TLR4 [106, 111].

### 6.2. Targeting R4 by MEM-18 Antibodies Suppresses LBP·LPS Binding to CD14

Anti-CD14^MEM-18^Fc^mIgG1^ antibody binds to L_51_–A_64_ sequence in R4 region (D_57_–A_64_) of CD14. It is able to interfere with entry of lipid A, the hydrophobic region of LPS, into the hydrophobic pocket of CD14 (R4) during CD14 recognition of LBP·LPS, thereby suppressing the harmful effects caused by LPS [113–116]. Thus, despite the differences in isotype, the anti-CD14^MEM-18^Fc^mIgG1^ antibody, similar to anti-CD14^3C10^Fc^mIgG2b^ antibody, prevents the binding of LPS to CD14 [113, 117]. As a result, LPS-induced production of both TNF-*α* from human monocytes [97, 118] or IL-8 from PBMC [119] has been suppressed. The effectiveness of anti-CD14^MEM-18^Fc^mIgG1^ antibody can be explained also by its supplementary ability to downregulate CD14 and TLR4, but not CD11b/CD18, from the cell surface as has been shown earlier using differentiated monocytic THP-1 cells [113, 120]. Since anti-CD14^MEM-18^ is a mIgG1 antibody and may be recognized by Fc*γ*Rs (CD14 ← anti-CD14^MEM-18^Fc^mIgG1^ → CD64/CD32), its mode of action is more complicated [113, 121].

It is necessary to note that LPS-induced IL-8 production has been shown to be suppressed more effectively by anti-CD14^MEM-18^ antibody than anti-TLR4^HTA125^Fc^mIgG2a^ antibody [119]. The effectiveness of anti-CD14^UCHM-1^Fc^mIgG2a^ antibody against LPS-induced effects in human monocytes is less evident than that of anti-CD14^MEM-18^Fc^mIgG1^ antibody [97, 118].

### 6.3. Targeting R3 by MY4 Antibodies Causes Internalization of CD14 and TLR4

Anti-CD14^MY4^Fc^mIgG2b^ antibody binds to S_34_–G_44_ sequence of CD14 and does not prime human PMNs for fMLP-triggered O_2_^·−^/ROS production (reduction of ferricytochrome C) [104, 113] but suppresses LBP·LPS-induced CD11b/CD18 mobilization to the cell surface [87]. Moreover, both LPS-induced association of G_i*α*2_ with PMN plasma membrane and activation of PLD are significantly suppressed by prior cell exposure to anti-CD14^MY4^ antibody [104]. The effectiveness of anti-CD14^MY4^Fc^mIgG2b^ antibody against LPS-induced effects is associated with its ability to induce downregulation of CD14 and TLR4 from the cell surface. It is interesting to note that LPS-independent internalization of CD14 and TLR4 during cell response to anti-CD14^MY4^Fc^mIgG2b^ antibody exceeded that of anti-CD14^MEM-18^Fc^mIgG1^ antibody [100, 104, 113]. Thus, the effectiveness of anti-CD14^MY4^ antibody against LPS-induced effects is based on its ability to block LBP·LPS binding to CD14 and to downregulate CD14 and TLR4 from the cell surface. Why mIgG2b antibodies to CD14 (MY4) or TLR4 (HT4) are internalized better than mIgG1 anti-CD14^MEM-18^ antibody remain to be elucidated.

### 6.4. Anti-CD14 Antibodies as a Constituent of Therapeutic Medications

CD14 as evidenced from data presented in Table 1 is involved in LPS-dependent PMN priming [103]. The relative weak effectiveness of anti-CD14^UCHM-1^Fc^mIgG2a^ antibody as a suppressor of LPS-dependent PMN priming for fMLP-triggered O_2_^·−^/ROS production may be explained by its epitope specificity that blocks CD14 incompletely [14]. However, the inhibitory effectiveness of anti-CD14^UCHM-1^ antibodies may be improved by replacing their mIgG2a isotype with mouse or human IgG1.

The therapeutic relevance of anti-CD14^28C^Fc^mIgG1^ or anti-CD14^18E12^Fc^mIgG1^ antibodies against LPS-induced effects has been already studied *in vivo* in INF-*γ*-sensitized *Macaca fascicularis* [109] and in normal human subjects (anti-CD14^IC14^Fc^mhIgG1^) [122]. Anti-CD14^28C/18E12^Fc^mIgG1^ antibodies protect primates from most of the physiologic and proinflammatory consequences of acute endotoxemia. The intravenous treatment of *M. fascicularis* by anti-CD14^18E12^ antibody blocks signaling events without affecting the binding of LPS to CD14 as it has been estimated during LPS-induced production of TNF-*α*. On the other hand, productions of IL-6 and IL-1*β* have been inhibited better by another anti-CD14^28C^ antibody that is able to block LBP·LPS binding to CD14 [109]. A beneficial anti-CD14^IC14^Fc^mhIgG1^ antibody attenuates acute LPS-induced clinical symptoms and strongly inhibits LPS-induced production of proinflammatory cytokines, while it only delayed the release of the anti-inflammatory cytokines such as soluble TNF receptor type I and IL-1 receptor antagonist [122].

## 7. Epitope Specificity and Effectiveness of Anti-TLR4 Antibodies against LPS-Induced Effects

Human TLR4 is linked to a range of diseases, including infectious disease, atherosclerosis, asthma, cardiac disease, liver disease, renal disease, inflammatory bowel disease, obesity, diabetes (types I and II), rheumatoid arthritis, Alzheimer's disease, Parkinson's disease, and multiple sclerosis [1, 6, 61, 123]. As a result, targeting TLR4 has attracted increasing attention in the context of anti-inflammatory medications for patients with different TLR4-dependent complications [6, 15, 16, 62, 106, 124–129]. The data presented in Table 2 represent TLR4 as a promising therapeutic target for “antibody”-based therapy.

The extracellular region of TLR4 can be divided into N-terminal (L_52_–P_202_), central (L_203_–L_348_), and C-terminal (K_349_–F_582_) domains; each of which contains LRRs 1–6, LRRs 7–12, and LRRs 13–22, respectively [1].

### 7.1. Targeting the N-Terminal Domain of TLR4

Targeting LRR2–LRR7 (D_50_–I_190_) repeats in TLR4 by anti-TLR4^HTA125^Fc^mIgG2a^ antibody does not suppress LPS-dependent PMN priming for fMLP-triggered O_2_^·−^/ROS production (luminol) [12, 132]. The same result has been obtained by Sanui et al. [133]. These authors did not observe pronounced inhibitory effect of anti-TLR4^HTA125^ antibody on LPS-induced PMN priming. However, Stadlbauer et al. [129] showed that the anti-TLR4^HTA125^ antibody is able to suppress LPS-induced production of ROS (Phagoburst kit) in PMNs.

When LPS-induced production of IL-6 and IL-8 was studied in the human embryonic cell line HEK293, no inhibitory effect of anti-TLR4^HTA125^Fc^mIgG2a^ was detected [6, 11]. However, in human retinal pigment epithelial cells, anti-TLR4^HTA125^ antibody was almost equally as effective as anti-CD14^UCHM-1^Fc^mIgG2a^ antibody in suppression of LPS-induced IL-8 production. It is interesting to note that simultaneous use of anti-TLR4^HTA125^ and anti-CD14^UCHM-1^ antibodies did not further potentiate antibody inhibitory effectiveness, suggesting that blockage of initial LPS binding to CD14 was highly effective and not further increased when TLR4 was also targeted [110].

The weak inhibitory effectiveness of anti-TLR4^HTA125^Fc^mIgG2a^ antibody could be explained by the epitope specificity. This antibody recognizes an antigenic epitope within D_50_–I_190_ sequence and binds to TLR4 irrespective of the presence or absence of MD-2 [132]. Thus, it may be suggested that blockage of LRR2–LRR7 (HTA125) is not enough to prevent LPS·MD-2-induced TLR4 dimerization (Table 2).

### 7.2. Targeting the C-Terminal Domain of TLR4

Anti-TLR4^HT4^Fc^mIgG2b^ antibody recognizes the nonlinear epitope within the LRR13 repeat of TLR4. This epitope is composed of several amino acid residues (K_349_, K_351_S_352_, G_364_NA, and S_368_E) closely located to the TLR4 dimerization interface created by LRR15–LRR17 repeats of two LPS·MD-2·TLR4 complexes. Based on experimental data, it has been assumed that anti-TLR4^HT4^ antibody is unable to prevent LPS·MD-2 binding to TLR4 but nevertheless inhibits LPS·MD-2-induced TLR4 internalization. The effectiveness of anti-TLR4^HT4^Fc^mIgG2b^ antibody in suppression of lipid A-induced production of TNF-*α*, IL-6, and IL-12p40 from human leukocytes is better than that of the anti-TLR4^HTA125^Fc^mIgG2a^ antibody [1, 5, 6]. Taking these facts into consideration, it could be concluded that targeting the C-terminal domain of TLR4 may lead to a more pronounced therapeutic effect than targeting the N-terminal domain of TLR4 by HTA125^mIgG2a^ or HT52^mIgG1^ antibodies. The effect of anti-TLR4^HT52^Fc^mIgG1^ (LRR2–LRR7: D_50_–I_190_ sequence) antibody may be potentiated by simultaneous application with anti-TLR4^HT4^Fc^mIgG2b^ (LRR13) antibody thus causing double blocking of TLR4 (LRR2–LRR7 and LRR13) [1, 5, 6]. Note that anti-TLR4^HTA125^ antibody recognizes the same antigenic epitope as did anti-TLR4^HT52^ antibody. We assume that double targeting TLR4 by anti-TLR4^HT4^ and anti-TLR4^HTA125^ antibodies would improve the inhibitory effectiveness of the latter.

As has been shown experimentally, the inhibitory effectiveness of anti-TLR4^HT4^Fc^mIgG2b^ or anti-TLR4^HT52^Fc^mIgG1^ is not associated with engagement of Fc*γ*Rs. In fact, the inhibitory effectiveness of anti-TLR4^HT4^Fc^mIgG2b^ or anti-TLR4^HT52^Fc^mIgG1^ against lipid A-induced effects was unaffected by prior cell exposure to blocking anti-CD32^AT-10^Fc^mIgG1^ antibody [1, 5, 6].

### 7.3. Targeting Both N- and C-Terminal Domains of TLR4

The improved antibody effectiveness seen with double-targeted TLR4 led to the generation of a new anti-TLR4^15C1^Fc^mIgG1^ antibody recognizing both LRR12 (Y_328_N) and LRR13 (K_349_LK, E_369_VD) sequences [11]. Anti-TLR4^15C1^ antibody blocks TLR4 binding to LPS·MD-2 and TLR4 dimerization as well. In addition, anti-TLR4^15C1^ antibody effectively suppresses LPS-induced IL-6 and IL-8 production analogous to anti-TLR4^HT4^Fc^mIgG2b^ or anti-TLR4^HT52^Fc^mIgG1^ antibodies but with stronger effect than anti-TLR4^HTA125^Fc^mIgG2a^ antibody [6, 11]. Furthermore, anti-TLR4^15C1^ antibody prevents LPS-induced TLR4 partitioning into lipid rafts [61]. As earlier has been shown, the effectiveness of anti-TLR4^15C1^Fc^mIgG1^ antibody is dependent on the engagement of Fc*γ*Rs (CD32) [1, 11]. Targeting CD32 by anti-CD32^IV.3/AT-10^Fc^mIgG2b^ antibodies dramatically reduces the effectiveness of anti-TLR4^15C1^Fc^mIgG1^ antibody when LPS-induced production of IL-6 was studied. It is necessary to note that LPS-induced IL-6 production had not been significantly affected by isotype-matched control mIgG1. Thus, in addition to engagement of CD32 (TLR4 ← anti-TLR4^15C1^Fc^mIgG1^ → CD32) and its signaling pathway(s), the therapeutic effect of anti-TLR4^15C1^Fc^mIgG1^ antibody is based on its ability to prevent LPS·MD-2 binding to TLR4 thereby abolishing TLR4 dimerization and its movement into lipid rafts [1, 11, 134].

### 7.4. Humanized Anti-TLR4 Antibody and Fc*γ*Rs

The differences in the affinity of Fc*γ*Rs for IgG subclasses have been explored in development of new therapeutic antibodies such as Hu15C1. This antibody is the humanized version of anti-TLR4^15C1^Fc^mIgG1^ antibody [61, 135]. Two substitutions (N_325_ → S and L_328_ → F) have been introduced into Fc arm of anti-TLR4^15C1^Fc^mIgG1^ antibody to amplify its inhibitory effectiveness. As a result, the affinity of the new anti-TLR4^Hu15C1^Fc^hIgG1^ for CD64 is potentiated, while for CD16, it is eliminated. An intermediate affinity of anti-TLR4^Hu15C1^Fc^hIgG1^ for CD32 was detected. Thus, CD64 (TLR4 ← anti-TLR4^Hu15C1^Fc^hIgG1^ → CD64/CD32) is viewed as the first contributor to the potent inhibitory effectiveness of anti-TLR4^Hu15C1^Fc^hIgG1^ antibody. In addition, CD32-initiated ITAMi signaling is expected when CD64 would be not available. The blockage of CD32B does not change significantly the inhibitory effectiveness of anti-TLR4^Hu15C1^ antibody. When anti-TLR4^Hu15C1^ antibody had been compared with the parental anti-TLR4^15C1^Fc^mIgG1^ antibody, the former antibody was more effective than the latter in inhibition of LPS-induced effects. In addition, it has been shown using neuronal originated HEK293 cells that CD32A is involved in the inhibitory potency of anti-TLR4^Hu15C1^ antibody. It was also discovered that the inhibitory potency of anti-TLR4^Hu15C1^ antibody against LPS-induced effects is much higher in CD32A-positive HEK293 cells than in CD32A-negative ones. Besides engagement of CD64 and CD32, the anti-TLR4^Hu15C1^ antibody is able to interfere with LPS-induced TLR4 dimerization thereby preventing TLR4 partitioning into lipid rafts. Thus, it is postulated that dimerization of TLR4 is a prerequisite for TLR4 clustering [61].

The therapeutic effectiveness of humanized anti-TLR4^NI-0101^ antibody has been already evaluated *ex vivo* and *in vivo* in the absence or presence of systemic LPS challenge (acute inflammation) [7, 9]. The other effective neutralizing anti-TLR4^WN1222-5^ antibody mimics the site of TLR4, recognizing an inner core structure of LPS from most infectious bacteria regardless of the presence of O-polysaccharide (O-antigen) and prevents LPS binding to target cells in the bloodstream [136].

### 7.5. Impact of CD32 in Effectiveness of IgG Antibodies

Antigenic recognition of CD32A by anti-CD32^IV.3^ Fab/F(ab′)_2_ fragments recognizing the F_132_SHLDP_137_ sequence does not cause Ca^2+^ mobilization, upregulation of p38 and ERK MAPK kinases, and intracellular production of H_2_O_2_/ROS (scopoletin) [137]. The other anti-CD32^CIKM5^ Fab/F(ab′)_2_ are also unable to induce Ca^2+^ mobilization when crosslinking by secondary F(ab′)_2_ fragments is omitted [64, 78]. Targeting CD32 by anti-CD32^AT-10^ F(ab′)_2_ inhibits constitutive and fMLP-triggered O_2_^·−^/ROS (luminol) production by PMNs [134, 138]. In PMNs, a small rise in intracellular free Ca^2+^ has been induced also by anti-CD32^CIKM5^Fc^mIgG1^ antibody but it was not associated with O_2_^·−^ release (SOD-inhibitable reduction of ferricytochrome C) [64, 78]. Antigenic recognition of CD32A by anti-CD32^AT-10^ Fab fragments recognizing an epitope located near or within IgG-binding site fails to activate differentiated monocytic THP-1 cells [134, 138]. Recent findings however have indicated that antigenic CD32 recognition by anti-CD32^IV.3/AT-10^ Fab/F(ab′)_2_ induced ITAMi signaling [134]. It has been shown that engagement of CD32 by anti-CD32^AT-10^ F(ab′)_2_ stimulates transient recruitment of tyrosine kinase ^2SH2^Syk/p72^Tyr^ to cytoplasmic domain of CD32A followed by incomplete phosphorylation of the ITAM leading to the inhibitory ITAMi conformation. As a result, activated ITAMi allows tyrosine phosphorylation of SHP-1 (protein tyrosine phosphatase) followed by inhibition of the major intracellular signaling players of immune cells such as guanine nucleotide exchange factor Vav-1 (RacGEF) and IRAK-1 kinase that are both involved in O_2_^·−^/ROS and cytokine production. Thus, blockage of Vav-1 in human PMNs can abrogate association of p67^phox^ with NADPH oxidase thereby suppressing O_2_^·−^/ROS production [134]. This suppressive effect of CD32A and mIgG1 might be reversed by LPS-induced assembly and stabilization of TLR4, CD11b/CD18, and Fc*γ*Rs in lipid rafts followed by the activation of classical ITAM signaling [11, 41, 44, 79, 139]. Since PMNs express very low levels of CD32B, an impact of its involvement in the inhibitory effectiveness of mIgG1 antibodies should be negligible [140].

Antigenic recognition of CD16 by anti-CD16^3G8^ Fab/F(ab′)_2_ despite the minor rise in free Ca^2+^ does not cause activation of p38 and ERK MAPK kinases nor actin polymerization and intracellular H_2_O_2_/ROS production (DHR) [137, 141, 142]. In addition, fMLP-triggered ROS production (luminol) is also not influenced by prior PMN exposure to anti-CD16^3G8^ F(ab′)_2_ [143]. Thus, it can be concluded that targeting CD16 by anti-CD16^3G8^ Fab/F(ab′)_2_ is unable to prime or upregulate intracellular O_2_^·−^/ROS production in PMNs. By contrast, in monocytes, antibody-dependent association of CD32 with CD16 (CD16 ← anti-CD16^3G8^Fc^mIgG1^ → CD32) in the plane of plasma membrane is able to initiate intracellular signaling events leading to generation of Ins(1,4,5)P_3_, DAG, and Ca^2+^ mobilization but not to calcium influx [64, 78].

Now, the impact of mIgG subclasses in activation of human immune cells has been revealed. It has been shown in PMNs that mIgG2a is unable to induce protein tyrosine phosphorylation and substantial rise in intracellular free Ca^2+^ [70, 144]. When we have used mIgG1 or mIgG2a and unprimed PMNs, only marginal fMLP-triggered O_2_^·−^/ROS production was observed [12–14]. Neither anti-CD11b^60.1/44^Fc^mIgG1^ nor anti-CD11b/CD18^IB4^Fc^mIgG2a^ antibodies are not able to induce Ca^2+^ mobilization in PMNs [70, 145, 146]. Targeting CD11b/CD18 on freshly prepared PMNs by anti-CD11b^Leu-15^Fc^mIgG2a^ antibody does not stimulate considerable intracellular O_2_^·−^/ROS production [62, 63, 147]. Anti-CD11b/CD18^IB4^Fc^mIgG2a^ or anti-CD11b^44a^Fc^mIgG1^ antibodies cause negligible intracellular H_2_O_2_/ROS (DHR) production in PMNs and even to a lesser extent than agonistic anti-CD11b^VIM12^Fc^mIgG1^ antibody [147]. Antibody-dependent association of CD11b/CD18 with CD64/CD32 (CD11b ← anti-CD11b^Leu-15^Fc^mIgG2a^ → CD64/CD32 or CD11b ← anti-CD11b^44a^Fc^mIgG1^ → CD32 or CD11b/CD18 ← anti-CD11b/CD18^IB4^Fc^mIgG2a^ → CD64/CD32) is also unable to stimulate considerable intracellular O_2_^·−^/ROS production in PMNs. These data, including ours, may suggest that neither mIgG2a nor mIgG1 are able to stimulate substantial rise in intracellular free Ca^2+^ and ROS production in freshly isolated PMNs. It is necessary to note that agonistic anti-CD11b^VIM12^Fc^mIgG1^ antibody recognizing the CD11b lectin site causes intracellular H_2_O_2_/ROS production in PMNs to the same extent as fMLP [147].

By contrast, in macrophage-like Mono Mac 6 cells, LPS-induced production of TNF-*α* and IL-10 has been potentiated by mIgG2a, while production of IL-1*β* has been suppressed [62]. A line of other data indicates that LPS-induced activation of IRAK and production of TNF-*α* and IL-8 from differentiated human monocytic THP-1 cells are not influenced by mIgG2a [106, 124, 127].

### 7.6. Upregulation of PMN Sensitivity to mIgG2a after IFN-*γ* Priming

IFN-*γ* is the most potent priming agent released in bloodstream during LPS-induced inflammation [15, 16, 148, 149]. Thus, during inflammation, the expression of CD64 on PMN is upregulated, while CD32 is unaffected or downregulated [62, 63]. As a result, IFN-*γ*-primed PMNs (20 h) acquire the ability to respond to mIgG2a antibodies such as anti-CD11b^Leu-15^Fc^mIgG2a^ by marked intracellular O_2_^·−^/ROS production (DCFDA) (CD11b ← anti-CD11b^Leu-15^Fc^mIgG2a^ → CD64) [63]. However, IFN-*γ* does not confer on PMNs the capability to generate O_2_^·−^/ROS in response to mIgG1 antibodies such as anti-CD11b^VIM12^Fc^mIgG1^ or anti-CD11b^5A4.C5^Fc^mIgG1^ (CD11b ← anti-CD11b^VIM12/5A4.C5^Fc^mIgG1^ → CD32), which is in agreement with IFN-*γ*-dependent CD32 downregulation [62, 63]. Thus, we concluded that only primed PMNs would be sensitive to mIgG2a antibodies, while freshly isolated PMNs are marginally responsive to mouse antibodies with IgG2a or IgG1 isotypes (regardless of epitope specificity).

In humans, as it has been revealed *in vivo*, the LPS administration causes an initial rapid decline in the absolute PMN counts at 1 h followed by an increase that reaches a maximum at 6 h and then declines at 22 h to basal levels. In addition, LPS injection induces biphasic CD64 upregulation on circulating PMNs. The first phase has been observed after 1 h of LPS challenge, while the second started at 6 h and reached a maximum at 22 h [148].

By contrast, freshly isolated monocytes constitutively express high levels of surface CD64 [61, 62] and respond to anti-CD11b^VIM12^Fc^mIgG1^ or anti-CD11b^Leu-15^Fc^mIgG2a^ antibodies by intracellular ROS production (DCFDA). For example, anti-CD11b^Leu-15^Fc^mIgG2a^ antibody causes an increase in ROS production in monocytes of all responders, while agonistic anti-CD11b^VIM12^Fc^mIgG1^ in 60% only. When anti-CD11b^VIM12^Fc^mIgG1^ antibody has been devoid of the Fc arms, its ability to stimulate ROS (DCFDA) production in freshly isolated monocytes was diminished [63]. Thus, the agonistic activity of anti-CD11b^VIM12^Fc^mIgG1^ antibody represents the cumulative effect of epitope specificity and capability to engage CD64/CD32 receptors.

The engagement of CD64, and CD32 to a lesser extent, by anti-TLR4^HTA125^Fc^mIgG2a^ antibody has been already revealed using macrophage-like Mono Mac 6 cells (CD32 > CD64). Mouse IgG2a inhibits most effectively the binding of anti-TLR4^HTA125^Fc^mIgG2a^ antibody to Mono Mac 6 cells, while mIgG1 or mIgG2b reveals no significant effect. Further, the binding of anti-TLR4^HTA125^ antibody to Mono Mac 6 cells has been most effectively prevented by anti-CD64^10.1^Fc^mIgG1^ antibody by contrast to anti-CD32^FLI8.26^Fc^mIgG2b^ antibody. Targeting CD16 by anti-CD16^3G8^Fc^mIgG1^ antibody does not influence significantly on anti-TLR4^HTA125^ binding to Mono Mac 6 cells [62]. Based on these findings, the high affinity of human CD64 and moderate affinity of human CD32 for the constant Fc arm of mIgG2a antibodies can be concluded [62, 63]. These results indicate also that specific Fab or F(ab′)_2_ fragments against cell surface receptors would be most appropriate and safe for applying in “antibody”-based therapy.

In summary, the major mechanisms underlying the inhibitory effectiveness of anti-TLR4 antibodies follow (1) an interference with TLR4 binding to CD14·LPS and LPS·MD-2, (2) inhibition of ligand- (LPS-) induced conformational changes that are indispensable for TLR4 signaling (HT4, HT52, 15C1, and Hu15C1) [1, 11, 61], (3) prevention of ligand-induced TLR4 partitioning into lipid rafts and its subsequent internalization (HT4, HT52, and Hu15C1) [1], and (4) engagement of Fc*γ*Rs (CD32/CD64) followed by ITAMi-initiated inhibitory signaling interfering with positive signaling induced by other receptors on the same cell (CD14, MD-2·TLR4, and CD11b/CD18) [61, 134].

## 8. CD11b/CD18 and Their Signaling Partners

Macrophage-1 antigen (Mac-1, *α*_M_*β*_2_, or CD11b/CD18) is a complement receptor (CR3). It consists of noncovalently linked CD11b (integrin *α*_M_) and CD18 (integrin *β*_2_) subunits. Integrins regulate important leukocyte functions including adhesion, migration, proteolysis, phagocytosis, and oxidative (respiratory) burst [150]. In resting PMNs, integrins are maintained in a conformationally inactive state and are unable to bind their ligands [151]. On cell stimulation, “inside-out” signaling originating from nonintegrin cell surface receptors such as FPR1, Fc*γ*Rs, or TLR4 leads to vast conformational changes in CD11b/CD18, but not directly to receptor clustering (integrin redistribution in the plane of plasma membrane) [42, 52]. Thus, only “inside-out” primed integrins exhibit increased ligand-binding avidity and initiate “outside-in” signaling by themselves. CD11b/CD18 has been detected in the LPS-induced “receptor cluster” on monocytes and can act as a signaling partner for such receptors as FPR1 and CD14 [44, 106, 150, 152, 153]. CD11b/CD18 can be found also in association with Fc*γ*Rs, but the consequences of these functional interactions are not fully understood. The functional association of the GPI-anchored form of CD16 (^PMN^CD16B^GPI^) with CD11b/CD18 is mediated by the lectin-binding site of the latter [147, 151, 153–155]. The mechanisms by which CD11b/CD18 regulates leukocyte functions such as respiratory burst are still poorly understood.

Like other *β*_2_ integrins, CD11b consists of a short cytoplasmic tail, single transmembrane domain, and long extracellular domain. The extracellular domain of CD11b is composed of seven repeats. The V to VII repeats are similar to the divalent cation-binding “EF-hand” motif. The II and III repeats are separated by the I (inserted) domain that is known also as *α*A/I-domain [156]. The effectiveness of anti-CD11b/CD18 antibodies against LPS-induced effects is listed in Table 3.

### 8.1. Targeting the CD11b *α*A/I-Domain

Antigenic recognition of CD11b/CD18 by anti-CD11b^44^ Fab recognizing *α*A/I-domain does not lead to considerable changes in conformation of CD11b/CD18 nor to activation of “outside-in” signaling [161, 162]. However, full anti-CD11b^44a^ or anti-CD11b/CD18^IB4^Fc^mIgG2a^ antibodies induce epitope exposition by CD11b that is recognized by anti-CD11b^VIM12^Fc^mIgG1^ antibody [147]. This effect may be explained by antibody-dependent association of CD11b/CD18 with Fc*γ*Rs. So, 44a or IB4 antibodies are able to induce “outside-in” signaling followed by “inside-out” signaling leading to further conformational changes in CD11b/CD18.

### 8.2. Targeting the C-Terminal Lectin Domain of CD11b

Antigenic recognition of the C-terminal lectin domain of CD11b (AA_614–682_) located near the cell membrane by anti-CD11b^OKM1^ Fab or F(ab′)_2_ does not induce PMN intracellular H_2_O_2_/ROS (scopoletin) production [137]. In PMNs, neither anti-CD11b^60.1/44^Fc^mIgG1^ nor anti-CD11b/CD18^IB4^Fc^mIgG2a^ antibodies cause Ca^2+^ mobilization [70, 145, 146]. The same result was obtained during cell response to anti-CD18^MHM23^Fc^mIgG1^ or anti-CD11b/CD18^60.3^Fc^mIgG2a^ antibodies regardless of isotype differences [78, 145]. Further, intracellular H_2_O_2_/ROS (DHR) triggered in PMNs by antibody-dependent association of CD32 with CD11b/CD18 (CD11b/CD18 ← anti-CD11b^44/IB4^Fc^mIgG1/mIgG2a^) was less pronounced than that in response to agonistic anti-CD11b^VIM12^Fc^mIgG1^ antibody [147]. Thus, it can be concluded that simple antibody-dependent association of CD11b/CD18 with CD32 (regardless of epitope specificity) is not enough to initiate significant agonistic (activating) signaling events and H_2_O_2_/ROS production in PMNs, whereas to induce sufficient intracellular signaling by IgG1 antibodies, targeting of a particular epitope on CD11b is required. In fact, when agonistic anti-CD11b^VIM12^Fc^mIgG1^ antibody recognizing the CD11b lectin site had been employed to PMNs, “outside-in” signaling, clustering of activated CD11b/CD18, upregulation of PI3K and PKB/Akt signaling pathways, Ca^2+^ mobilization, and actin polymerization were all realized, but the Raf → MEK1/2 → ERK1/2 signaling pathway was not upregulated [163]. Agonistic anti-CD11b^VIM12^Fc^mIgG1^ antibody prevents association of CD11b/CD18 with CD16B^GPI^ [163] but nevertheless induces PMNs for generation of H_2_O_2_/ROS (DHR) to the same extent as fMLP. Anti-CD11b^VIM12^Fc^mIgG1^-dependent production of H_2_O_2_/ROS (DHR) exceeds that induced by IB4 or 44a antibodies [147]. Taking these facts into consideration, it may be suggested that CD11b/CD18-dependent “outside-in” signaling would be successfully realized when CD11b lectin domain is docked (thus, CD11b/CD18 is primed) by other appropriate GPI-anchored protein on the same cell. In PMNs, “outside-in” signaling is also initiated by CD11b/CD18 that has been clustered in the response to anti-CD11b^2LPM19c^ F(ab′)_2_ recognizing *α*A/I-domain. The same effect had been observed when whole anti-CD11b^2LPM19c^Fc^mIgG1^ antibody was used. Unexpectedly, monovalent anti-CD11b^2LPM19c^ Fab is unable to produce such agonistic activity [158]. These results clearly show that agonistic activity of anti-CD11b antibodies is determined by F(ab′)_2_ epitope specificity. Moreover, F(ab′)_2_ can potentially bind two targets leading to close proximity of two integrin molecules that mimic receptor crosslinking [164].

In PMNs, generation of H_2_O_2_/ROS (DHR) induced by anti-CD11b^VIM12^Fc^mIgG1^ antibody is not diminished by prior cell exposure to anti-CD11b^44a^Fc^mIgG1^ or anti-CD11b/CD18^IB4^Fc^mIgG2a^ antibodies. It necessary to note that sequential treatment of PMNs in whole blood by anti-CD11b/CD18^IB4^Fc^mIgG2a^ and anti-CD11b^VIM12^Fc^mIgG1^ antibodies led to more pronounced generation of H_2_O_2_/ROS (DHR) than treatment with anti-CD11b^44a^Fc^mIgG1^ combined with anti-CD11b^VIM12^Fc^mIgG1^ antibody [147]. This result may be explained by the fact that anti-CD11b/CD18^IB4^Fc^mIgG2a^ antibody is able to potentiate the binding of anti-CD11b^VIM12^Fc^mIgG1^ antibody to CD11b/CD18 in addition to their ability to affect both subunits of CD11b/CD18. Thus, anti-CD11b/CD18^IB4^Fc^mIgG2a^ antibody, besides binding to CD11b/CD18, can also engage other *β*_2_ integrins via the common CD18 subunits (CD11a/CD18 and CD11c/CD18). Such broad targeting of *β*_2_ integrins could provide sufficient signals for intracellular H_2_O_2_/ROS generation (DHR). This conclusion is supported by the fact that antigenic recognition of CD11b/CD18 by anti-CD11b/CD18^IB4^ F(ab′)_2_ induces Ca^2+^ mobilization to a similar extent as whole anti-CD11b/CD18^IB4^Fc^mIgG2a^ antibody [165].

In PMNs, generation of ROS/H_2_O_2_ (DHR) induced by anti-CD11b^VIM12^Fc^mIgG1^ antibody can be blocked almost completely by prior cell exposure to anti-CD32^IV.3^Fc^mIgG2b^ antibody [160]. Thus, unlike monocytes, CD32 receptor on PMNs is involved in CD11b/CD18-induced generation of ROS/H_2_O_2_ (DHR) in the response to anti-CD11b^VIM12^Fc^mIgG1^ antibody (CD11b/CD18 ← anti-CD11b^VIM12^Fc^mIgG1^ → CD32). Therefore, it has been supposed that association of CD11b/CD18 with CD32 is required for ROS generation during PMN response to anti-CD11b^VIM12^Fc^mIgG1^ antibody [147]. Ortiz-Stern and Rosales [73] have shown that CD32 and CD11b/CD18 are uniformly distributed and not colocalized on the surface of unstimulated PMNs. A similar observation has been made for ^PMN^CD16B^GPI^ which is also uniformly distributed across the cell surface and is not colocalized with CD32 in unstimulated PMNs [73].

By contrast to PMNs, in monocytes, a close spatial proximity between CD11b/CD18 and CD32/CD64 has been suggested by Gadd et al. [63]. These authors have observed that anti-CD11b^VIM12^Fc^mIgG1^ antibody interferes sterically with anti-CD32^IV.2^Fc^mIgG2b^ antibody for binding to CD32 on monocytes, but not on PMNs [63]. Interestingly, in their study, anti-CD11b^VIM12^Fc^mIgG1^ antibody had been unable to induce intracellular ROS (DCFDA) generation in PMNs as they did in monocytes [63, 147].

### 8.3. Anti-CD11b/CD18 Antibodies in Therapeutic Medications

In the light of data presented here, the effectiveness of anti-CD11b/CD18 antibodies against LPS-induced effects should be discussed. It has been shown that CD11b/CD18 has a site recognizing LPS carbohydrates, namely, N-acetyl-*D*-glucosamine (GlcNAc) and mannose. In addition, CD11b/CD18 binds truncated LPS glycoforms such as Re-LPS [153–155]. Two putative LPS-binding sites within the CD18 *β*A region (AA_216–248_, _266–318_) had been proposed earlier [166]. It was found that Re-LPS from *Salmonella minnesota* is bound through cationic interactions by the *β*A_266–318_-exposed CD18 sequence, while another *β*A_216–248_ sequence probably utilizes other interactions like hydrophobic ones [166]. Thus, it is not excluded that 3-deoxy-D-*manno*-octulosonic acid (KDO), the inner core sugar of almost all LPS molecules, may be involved in LPS recognition by CD18 [153, 155, 159, 160]. However, neither LPS binding to PBMC nor LPS-induced PMN priming for fMLP-triggered ROS production has been blocked by anti-CD11b^OKM1^Fc^mIgG1^ antibody [91, 99, 100, 104]. A similar result has been obtained in our work [13] where we used anti-CD11b^ICRF44^Fc^mIgG1^ antibody. This antibody binds probably to *α*A/I-domain of CD11b (AA_201–217_, _245–261_) in a manner independent of inactive or active CD11b/CD18 state [158, 162, 167]. Thus, it may be concluded that targeting the *α*A/I-domain of CD11b by anti-CD11b^ICRF44^Fc^mIgG1^ or the CD11b lectin site by anti-CD11b^OKM1^Fc^mIgG1^ antibodies is unable to suppress significantly LPS-dependent PMN priming for fMLP-triggered O_2_^·−^/ROS production [13, 91, 99, 100, 104]. From the data presented here, we can, however, not exclude that other CD11b/CD18 sites might be involved in LPS recognition. The effectiveness of anti-CD11b^44^Fc^mIgG1^ antibodies as suppressors of LBP·LPS-dependent PMN priming for fMLP-triggered O_2_^·−^/ROS production is less pronounced than that of anti-CD14^3C10^Fc^mIgG2b^ antibody [90]. Thus, targeting CD14 by appropriate antibodies would be more effective in comparison to targeting CD11b/CD18 or TLR4. Moreover, it has been shown that CD11b/CD18 interacts more avidly with aggregated but not monomeric LPS and this interaction occurs even better in the absence of LBP [106, 168]. Taking these data into consideration, we concluded that CD11b/CD18 is not an appropriate target for “antibody”-based therapy even when anti-CD11b Fab/F(ab′)_2_ fragments are used.

## 9. Conclusions

In summary, several conclusions can be drawn. Neither mIgG2a nor mIgG1 are able to stimulate Ca^2+^ mobilization and ROS production in freshly isolated PMNs.

Sufficient signals for phosphoinositide breakdown could be induced in monocytes by CD14 association with CD64. Moreover, this heterotypic CD14 association with CD64/CD32 (anti-CD14^UCHM-1^Fc^mIgG2a^) or association of two CD32 receptors together (CD32 ← anti-CD32^CIKM5^Fc^mIgG1^ → CD32) leads to Ca^2+^ signaling in monocytes. The homotypic CD32 association is less effective in Ca^2+^ signaling than heterotypic association of CD14 with CD64. In spite of this, CD14 association with CD64 is not enough to trigger O_2_^·−^/ROS production in monocytes. Ca^2+^ signaling caused by CD64/CD32 engagement without crosslinking is weaker than that induced by fMLP.

In monocytes not only sufficient production of PI(4,5)P_2_ but also robust upregulation of intracellular H_2_O_2_/ROS is initiated in response to broad CD14 crosslinking by anti-CD14^mIgG1^ antibodies and secondary F(ab′)_2_. In PMNs, this effect of CD14 crosslinking is less pronounced. TLR4 crosslinking [anti-TLR4^76B351.1^Fc^mIgG2b^ plus F(ab′)_2_] does not have the same effect on PI(4,5)P_2_ production as CD14 crosslinking. Thus, PI(4,5)P_2_ generation is a specific response to CD14 crosslinking (clustering).

Antigenic recognition of CD32 by anti-CD32^IV.3/AT-10^ Fab/F(ab′)_2_ is already able to induce ITAMi signaling in PMNs thereby suppressing both constitutive and fMLP-triggered O_2_^·−^/ROS production. Antigenic recognition of CD16 by anti-CD16^3G8^ Fab/F(ab′)_2_ is unable to prime or activate PMNs for intracellular O_2_^·−^/ROS production.

In most cases in PMNs, targeting CD11b [Fab^44^] causes “outside-in” signaling and generation of intracellular H_2_O_2_/ROS [Fab/F(ab′)_2_^OKM1^]. Antigenic recognition of both CD11b/CD18 subunits by F(ab′)_2_^IB4^ stimulates Ca^2+^ mobilization in the similar extent as the whole IB4 antibody. In PMNs, antibody-dependent [anti-CD11b^mIgG1/mIgG2a^] association of CD11b/CD18 with CD32 or CD64 without crosslinking (clustering) does not induce sufficient Ca^2+^ mobilization and intracellular O_2_^·−^/ROS production. Thus, nonagonistic targeting CD11b followed by its association with CD32 is not enough to activate PMNs for significant H_2_O_2_/ROS generation but it takes place when CD11b will be targeted by agonistic antibody followed by CD32 engagement. A high sensitivity of CD11b/CD18 to environmental stimuli including antibodies and its ability to initiate “inside-out” signaling makes CD11b/CD18 not an appropriate target for “antibody”-based therapy even if anti-CD11b Fab/F(ab′)_2_ fragments would be used.

By contrast to CD11b/CD18, targeting CD14 by Fab or F(ab′)_2_ fragments is the most appropriate and safe approach for “antibody”-based therapy for LPS-induced deleterious effects even when innate immune cells are primed by PAMPs or endogenous priming molecules. A similar conclusion can be made accordingly targeting TLR4 by specific Fab/F(ab′)_2_ fragments. The effectiveness of anti-TLR4 Fab/F(ab′)_2_ fragments further may be potentiated by simultaneous use with anti-CD14 Fab/F(ab′)_2_. However, it is necessary to kept in mind that partial TLR4 blockage may cause the better therapeutic effect since certain TLR4 activation is required for development of the adaptive immune responses. Anti-TLR4 antibody is able to interfere with TLR4 binding to LPS·MD-2 thereby preventing TLR4 dimerization in addition to engagement of CD32 and CD64 signaling pathways.

In light of the data presented and based on our own observations [12, 13, 14], we can conclude that only anti-CD14^UCHM-1^Fc^mIgG2a^ but neither anti-TLR4^HTA125^Fc^mIgG2a^ nor anti-CD11b^ICRF44^Fc^mIgG1^ antibodies are able to prevent significantly LPS-induced PMN priming for fMLP-triggered O_2_^·−^/ROS generation. Therefore, we confirm that CD14 is really the main TLR4 gatekeeper. We believe that anti-CD14 Fab/F(ab′)_2_ fragments will be very suitable for clinical use and could improve outcomes during LPS-initiated inflammation.

## Figures and Tables

**Table 1:** The capability of antibodies against CD14 affects the LPS-induced effects (the references are indicated inside the square brackets).

Clone (isotype)	Epitope	Influence on LPS-induced effects		References
		Does	Does not	
*3C10* (mIgG2b) Effectiveness decreases when LPS concentration increases	E_7_–R_14_	(1) Suppress CD14 binding to LBP·Re-LPS *Salmonella minnesota* (1 ng/ml) as well as PMN priming for fMLP-triggered О_2_^·−^/ROS (2) Whole or F(ab′)_2_ suppress О_2_^·−^/ROS production in monocytes challenged by Re-LPS *Escherichia coli* (1 ng/ml, 5% blood serum) (3) Prevent CD11b/CD18 mobilization to the cell surface in PMNs stimulated by Ra/Rb-LPS *E. coli* K12 (30 ng/ml, without serum)		[90–96]
*biG10* (mIgG1)	D_9_–F_13_	(1) Suppress TNF-*α* production in whole human blood exposed to LPS *Salmonella abortus-equi* (10 ng/ml)		[97, 98]
*MY4* (mIgG2b)	S_34_–G_44_	(1) Suppress CD14 binding to LBP·Re-LPS (2) Decrease PMN priming by LPS from *E. coli* O55:B5 (10 ng/ml, 1% serum) or *E. coli* O111:B4 (10 ng/ml, 10% serum) (3) Inhibit phosphatidic acid generation in LPS-primed and fMLP-stimulated PMNs (4) Suppress LPS-dependent activation of p38 MAPK in human PMNs (5) Whole or Fab suppress LPS uptake by human monocytes	(1) Affect fMLP-triggered О_2_^·−^/ROS production from unprimed PMNs	[77, 94, 99–104]
*60bca* (mIgG1)	S_34_–V_38_	(1) Prevent LBP·Re-LPS *S. minnesota* binding to CD14 (2) Abolish almost completely PMN priming by LBP·Re-LPS *S. minnesota* (1 ng/ml) for fMLP-triggered О_2_^·−^/ROS		[90, 105]
*63D3* (mIgG1)		(1) Whole or F(ab′)_2_ suppress weakly LPS-induced ROS production in human monocytes	(1) Prevent LBP-dependent delivery of Re-LPS *S. minnesota* to CD14 (2) Suppress LPS-induced TNF-*α* and IL-8 production	[91, 94, 95, 106–108]
*28C5* (mIgG1)		(1) Suppress LBP-dependent delivery of Re-LPS *S. minnesota* to CD14 (2) Suppress LPS-dependent activation of p38 MAPK		[94, 109]
*biG14* (mIgG2a)	E_39_–G_44_	(1) Decrease binding of Ra-LPS *E. coli* to CD14		[97]
*UCHM-1* (mIgG2a)		(1) Suppress LPS-induced IL-8 production by human retinal pigment epithelial cells (2) Decrease PMN priming by S- or Re-LPS *E. coli* (100 ng/ml, 2% serum) for fMLP-triggered О_2_^·−^/ROS production		[14, 110]

**Table 2:** The capability of antibodies against TLR4 affects the LPS-induced effects (the references are indicated inside the square brackets).

Clone (isotype)	Epitope	Influence on LPS-induced effects		References
		Does	Does not	
*HTA125* (mIgG2a)	Within D_50_–I_190_ *LRR2–LRR7*	(1) Suppress by 30% the intracellular О_2_^·−^/ROS production in PMNs stimulated by LPS *E. coli* O111:B4 (100 ng/ml) (2) Suppress IRAK degradation induced by LPS *E. coli* O111:B4 (500 ng/ml, 10% FCS) in THP-1 (CD14^+^, CD11b/CD18^+^) cells (3) Inhibit by 80% NF-*κ*B activation in monocytes stimulated by O-4′ MPLA *E. coli* (10 *μ*g/ml) (4) Inhibit TNF-*α* (55%) and IL-10 (85%) production from PBMC stimulated by O-4′ MPLA *E. coli* (10 *μ*g/ml) (5) Suppress TNF-*α* production from differentiated THP-1 cells stimulated by LOS (1 ng/ml, without serum) from *Neisseria meningitidis* NMB	(1) Affect О_2_^·−^/ROS production caused in PBMC by Ra/Rb-LPS *E. coli* K12LCD25 (3 ng/ml) (2) Influence on PMN priming by S- or Re-LPS *E. coli* (100 ng/ml, 2% serum) for fMLP-triggered О_2_^·−^/ROS production	[12, 106, 119, 124, 125, 130, 129]
*HT52* (mIgG1)	Within D_50_–I_190_ *LRR2–LRR7*	(1) More effectively suppress IL-6 production induced in U373/TLR2^−^ cells by LPS *E. coli* (100 ng/ml) than HTA125 antibody does (2) Suppress TNF-*α* and IL-6 production in whole human blood stimulated by lipid A *E. coli* (10 ng/ml) in the same extent as HTA125 antibody does		[1, 5]
*15C1* (mIgG1)	Y_328_N (*LRR12*) K_349_LK and EVD_369_ (*LRR13*)	(1) Suppress IL-6 production in whole blood stimulated by Ra/Rb-LPS *E. coli* K12LCD25 (4 ng/ml)		[6, 11, 61]
*Hu15C1* (hIgG1)	Y_328_N (*LRR12*) K_349_LK and EVD_369_ (*LRR13*)	(1) Suppress IL-6 production from differentiated U937 cells stimulated by Re-LPS *S. minnesota* 595 (1 ng/ml)		[61]
*HT4* (mIgG2b)	K_349_LKS_352_ and G_364_NAFSE_369_ (*LRR13*)	(1) Suppress LPS-induced internalization of TLR4 (2) Probably stimulate dimerization of two TLR4	(1) Interfere with transfer of mCD14·LPS to MD-2·TLR4 and LPS-induced TLR4 dimerization as well	[6, 131]
*HTA405* (isotype not pointed out)		(1) Suppress production of TNF-*α* and IL-8 from THP-1/CD14^+^ cells stimulated by Re-LPS *S. minnesota* R595 (10 ng/ml) (2) The same positive effectiveness of HTA405 on TNF-*α* production has been observed in whole blood stimulated by Ra/Re-LPS *S. minnesota* or S/Re-LPS *E. coli* (3) Decrease IL-6 production by 50% or 20% from U373/TLR2^−^ cells stimulated by Re-LPS *S. minnesota* R595 (10 ng/ml) or S-LPS *E. coli* O111:B4 (100 ng/ml), respectively	(1) Marginally decrease the production of TNF-*α* and IL-8 from THP-1/CD14^+^ cells stimulated by S-LPS *E. coli* O111:B4 (100 ng/ml, 2% serum)	[108]
*HT414* *HTA1216* (isotypes not pointed out)		(1) Suppress production of TNF-*α* and IL-8 from THP-1/CD14^+^ cells stimulated by Re-LPS *S. minnesota* R595 (10 ng/ml)	(1) Marginally suppress the production of TNF-*α* and IL-8 from THP-1/CD14^+^ cells stimulated by S-LPS *E. coli* O111:B4 (100 ng/ml)	[108]

**Table 3:** The capability of antibodies against CD11b/CD18 affects the LPS-induced effects (the references are indicated inside the square brackets).

Clone (isotype)	Epitope	Influence on LPS-induced effects		References
		Does	Does not	
*OKM-1* (mIgG2b)	CD11b AA_614–682_ (lectin site)	(1) Increase TNF-*α* production from monocytes stimulated by LPS *S. minnesota* (1 ng/ml, without serum)	(1) Inhibit binding of LPS *E. coli* O111:B4 (10 ng/ml, 10% FCS) to human PBMC (2) Inhibit PMN priming by LPS *E. coli* O55:B5 (100 ng/ml, 1% serum) for fMLP-triggered О_2_^·−^/ROS production (3) Inhibit protein (41 and 42 kDa) tyrosine phosphorylation in macrophages stimulated by Re-LPS *S. minnesota* R595 (1 ng/ml, human serum) (4) Influence on О_2_^·−^/ROS production induced by Re-LPS *E. coli* (1 ng/ml, 5% serum) in human monocytes (5) Influence on TNF-*α* production from human PBMC stimulated by LPS *E. coli* O111:B4 (100 ng/ml, 10% serum)	[63, 91, 99, 100, 104, 105, 157, 158]
*904* (mIgG2b)	CD11b AA_74–316_ (*α*A/I-domain)	(1) Suppress macrophage interaction with bovine erythrocytes opsonized by Re-LPS *S. minnesota* R595		[159, 160]
*ICRF 44* (mIgG1)	CD11b		(1) Affect PMN priming by S- or Re-LPS *E. coli* (100 ng/ml, 2% serum) for fMLP-triggered О_2_^·−^/ROS production	[13]
*IB4* (mIgG2a)	CD11b/CD18	(1) Increase TNF-*α* production from monocytes stimulated by LPS *S. minnesota* (1 ng/ml, without serum)	(1) Inhibit binding of LPS *E. coli* O111:B4 (100 ng/ml, 10% serum) to human PBMC and TNF-*α* production (2) Influence on activation of p38 MAPK during PMN priming by LPS *E. coli* O55:B5 (5 ng/ml) for fMLP-triggered О_2_^·−^/ROS production	[77, 99, 157]

## Abbreviations

ADP: Adenosine diphosphate
ARF6: ADP-ribosylation factor 6
CD14: Glycosylphosphatidylinositol-anchored protein
DAG: Diacylglycerol
DAMPs: Damage-associated molecular patterns
ERK: Extracellularly regulated kinase
Fc*γ*Rs: Fc receptors *γ*
FPR1: Formyl peptide receptor 1
fMLP: *N*-Formyl-methionyl-leucyl-phenylalanine
GPCRs: G protein-coupled receptors
GPI: Glycosylphosphatidylinositol
GTPase: Guanine nucleotide triphosphatase
IC: Immune complexes
IL: Interleukin
INF-*γ*, *β*: Interferon *γ*, *β*
Ins(1,4,5)P_3_: Inositol 1,4,5-triphosphate
IRAK: IL-1 receptor-associated kinase
IRF3: Interferon regulatory factor 3
ITAM: Immunoreceptor tyrosine-based activation motif
ITIM: Immunoreceptor tyrosine-based inhibitory motif
LBP: Lipopolysaccharide-binding protein
LPS: Lipopolysaccharide (endotoxin)
LRR: Leucine rich repeat
MAPK: Mitogen-activated protein kinase
MCP-1: Monocyte chemoattractant protein 1
MD-2: Myeloid differentiation factor 2
MEK: Mitogen-activated ERK-activated kinase (also referred as MAP2K, MAPKK)
MIP-3*α*: Macrophage inflammatory protein 3*α*
MKK3: Mitogen-activated protein kinase kinase 3 (also referred as MAP2K3, MAPKK3, MEK3, and SAPKK-2)
MyD88: Myeloid factor of differentiation 88
NADPH oxidase: Nicotinamide adenine dinucleotide phosphate oxidase
NF-*κ*B: Nuclear factor *κ*-light-chain-enhancer of activated B cells
NOX4: NADPH oxidase complex 4
PAMPs: Pathogen-associated molecular patterns
PBMC: Peripheral blood mononuclear cells
PI3K: Phosphatidylinositol 3-kinase
PIP5K: Phosphatidylinositol 4-phosphate 5-kinase
PLC: Phospholipase C
PLD: Phospholipase D
PtdIns(3,4,5)P_3_ or PI(3,4,5)P_3_: Phosphatidylinositol 3,4,5-triphosphate
PtdIns(4,5)P_2_ or PI(4,5)P_2_: Phosphatidylinositol 4,5-bis-phosphate
PTK: Protein tyrosine kinase
PMNs: Polymorphonuclear leukocytes
PKB/Akt: Protein kinase B or serine/threonine-specific protein kinase alpha
Raf: Serine/threonine-protein kinase
ROS: Reactive oxygen species
SHP-1: Protein tyrosine phosphatase 1
SOD: Superoxide dismutase
TAK1: TGF-*β*-activated kinase
TGF-*β*: Transforming growth factor *β*
TNF-*α*: Tumor necrosis factor *α*
TICAM-1: TIR domain-containing adaptor molecule 1
TIR: Toll/interleukin-1 receptor domain
TIRAP/MAL: TIR domain-containing adaptor protein also known as MyD88 adaptor-like
TLR: Toll-like receptor
TRAF6: TNF-*α* receptor-associated factor
TRAM: TRIF-related adaptor molecule
TRIF: TIR domain-containing adaptor inducing interferon-beta
Vav-1/RacGEF: Guanine nucleotide exchange factor
VD3: 1*α*,25-Dihydroxyvitamin D3
DHR: Dihydrorhodamine 123
DCFDA: 2′,7′-Dichlorofluorescein diacetate
O-4′ MPLA: O-4′ monophosphorylated lipid A.
